# Impact of Hepatitis B Exposure on Sustained Virological Response Rates of Highly Viremic Chronic Hepatitis C Patients

**DOI:** 10.1155/2009/812140

**Published:** 2009-04-15

**Authors:** Ioannis S. Elefsiniotis, Christos Pavlidis, Elena Vezali, Theodoros Mariolis-Sapsakos, Sotirios Koutsounas, George Saroglou

**Affiliations:** ^1^University Department of Internal Medicine, Hepatology Unit, “Elena Venizelou” Hospital, 11521 Athens, Greece; ^2^Carchidonos 9, A. Glyfada, 16562, Greece; ^3^Reference Center for Viral Hepatitis, I.K.A, Athens, Greece

## Abstract

*Aim*. To evaluate the impact of hepatitis B core antibody (anti-HBc) seropositivity in sustained virological response (SVR) rates in treatment-naïve, chronic hepatitis C (CHC) patients with high pretreatment viral load (>800000 IU/mL). *Methods*. 185 consecutive CHC patients (14.4% cirrhotics, 70.2% prior intravenous drug users) treated with pegylated interferon-a2b plus ribavirin, for 24 or 48 weeks based on viral genotype, were retrospectively analyzed. SVR was confirmed by undetectable serum HCV-RNA six months after the end of treatment schedule. *Results*. Thirty percent of CHC/HBsAg-negative patients were anti-HBc-positive. Anti-HBc positivity was more prevalent in cirrhotic, compared to noncirrhotic patients (76.9% versus 19.5%, *P* < .05). Serum HBV-DNA was detected in the minority of anti-HBc-positive patients (1.97%). Overall, 62.1% of patients exhibited SVR, while 28.6% did not; 71.4% of non-SVRs were infected with genotype 1. In the univariate analysis, the anti-HBc positivity was negatively associated with treatment outcome (*P* = .065). In the multivariate model, only the advanced stage of liver disease (*P* = .015) and genotype-1 HCV infection (*P* = .003), but not anti-HBc-status (*P* = .726), proved to be independent predictors of non-SVR. *Conclusion*. Serum anti-HBc positivity does not affect the SVR rates in treatment-naïve CHC patients with high pretreatment viral load, receiving the currently approved combination treatment.

## 1. Introduction

The combination of interferon-alpha (IFNa) and ribavirin (RIB) produced response rates in approximately 40% of previously untreated patients with chronic hepatitis C [1, 2]. Pegylated interferon-alpha (PEG-IFNa) plus ribavirin is currently the treatment of choice in patients with chronic hepatitis C virus- (HCV-) related liver disease [3, 4]. Viral genotype, pretreatment viral load as well as patient age, body weight, and liver histopathology are important predictors of response according to landmark trials [4–7]. 

Hepatitis B virus (HBV) and HCV share the same routes of transmission but the vast majority of acutely infected patients recover spontaneously from HBV infection, whereas they become chronically infected by HCV, due to the different natural course of the viral infections [3, 8]. The clinical impact of occult HBV infection, defined as the presence of serum HBV-DNA in the absence of hepatitis B surface antigen (HBsAg) and its high prevalence in chronic HCV-infected patients has been documented by several studies [9–12]. Nevertheless, the significance of occult hepatitis B in terms of response to currently approved antiviral treatment of chronic hepatitis C patients, especially those with high pretreatment HCV viral load, remains controversial [13–16].

 The principal aim of our study was to evaluate the impact of anti-HBc seropositivity on the sustained virological response rates in treatment-naïve chronic hepatitis C patients with high pretreatment viral load, treated with PEG-IFNa2b plus RIB under real life conditions in Greece. Also, we intended to investigate the prevalence of detectable HBV-DNA in serum of anti-HBc-positive chronic hepatitis C patients of the study population.

## 2. Material and Methods

Two referral centers for chronic viral hepatitis in Greece participated in this study. One hundred eighty-five consecutive treatment-naïve patients with chronic hepatitis C (CHC) with available data from medical records from 2000 to 2007 were retrospectively analyzed. Inclusion criteria were detectable anti-HCV (ELISA III) at least two times within a year, detectable HCV-RNA in serum with a sensitive PCR assay and high pretreatment viral load (HCV-RNA > 800 IU/mL) within a month before the beginning of treatment, schedule, liver biopsy indicating chronic hepatitis within 6 months before treatment and elevated alanine aminotransferace (ALT) activity before treatment (>40 IU/L and <400 IU/L) and at least once during the last 6 months.

Exclusion criteria were decompensated liver disease, chronic liver disease of any other cause other than chronic HCV infection, and alcohol abuse (defined as consuming more than 30 gram alcohol per day for at least 5 years). Patients with HIV infection as well as active substance abuse were also excluded. 

All patients had been evaluated on admission clinically, hematologically, biochemically, and serologically, using routine commercially available methods. All participants of the study had their height and weight recorded at the beginning of the study, and their body mass index (BMI) had been calculated by dividing patient's weight (kg) by the squared height (m^2^). Serum HCV-RNA levels had been measured by a commercially available polymerase chain reaction (PCR) assay (Cobas Amplicor HCV test, version 2, Roche Diagnostics, Branchburg, NJ, USA) qualitatively (lower detection limit of 50 IU/mL) and quantitatively (range of detection 600–800 IU/mL). HCV genotype was performed using INNOLIPA HCV assay (Innogenetics, Belgium). Baseline stored serum samples of anti-HBc-positive patients of our study population were used in order to detect HBV-DNA, using a sensitive (lower limit of detection: 400 copies/mL), commercially available, PCR assay (Amplicor, HBV Monitor, Roche).

Liver biopsy had been performed at baseline; all biopsy specimens were evaluated by two experienced hepatopathologist and scored according to Ishak scoring system (grade = 0–18, stage = 0–6) [17].

All patients were treated with weight-based dosing of pegylated interferon-a2b (Peg-Intron, 1.5 *μ*g/kg/week) and genotype-based ribavirin dose (Rebetol, 800 mg/day for genotype 2/3 and 1000–1200 mg/day for genotype 1/4-infected patients, according to baseline body weight - < or ≥85 kg, resp.). The duration of treatment was 24 weeks for genotype 2/3-infected patients and 48 weeks for genotype 1/4-infected ones.

 Serum HCV-RNA data were available before the initiation of treatment (qualitative and quantitative PCR assay) and six months after the end of treatment schedule (qualitative PCR assay). Sustained virological response (SVR) was confirmed by undetectable serum HCV-RNA six months after completion of treatment. Patients with detectable serum HCV-RNA six months after treatment discontinuation were characterized as non-SVRs.

### 2.1. Statistical Analyses

The association of each genotype group with continuous variables was investigated with either ANOVA (using post-hoc Scheffe's test when omnibus test was significant) or Student's *t*-test. The association of each genotype group with categorical variables was explored with chi-square analyses. The association of SVR with continuous variables was investigated with Student's *t*-test and with categorical variables with chi-square analyses.

We used logistic regression analyses with SVR as the dichotomous dependent variable. In multivariate modes, in addition to genotype variable (which was consider the main predictor) we simultaneously included to the logistic regression patient's age, gender, BMI, anti-HBc serological status, grade and stage of liver disease. In order to further investigate possible age effects in the impact of the proposed predictors of SVR, we recalculated the logistic regression models, categorizing the patients of our study population with available data in three subgroups (<35 years/*n* = 54, 35–55 years/*n* = 75, >55 years/*n* = 56).

Local Hospital Institutional Review Board reviewed and approved this project. The study conformed to the ethical guidelines of the 1975 Declaration of Helsinki. Written informed concern of the study population was not necessary because this retrospective study did not modify the existing diagnosis or the therapeutic strategy.

## 3. Results

Epidemiological, serological, virological, and histological data of our study population are presented in Table 1. Low-grade HBV viremia (1280 copies/mL) was detected in one anti-HBc-positive/anti-HBs-negative cirrhotic patient (1.97% of the anti-HBc-positive study population). It is important to note that among 26 cirrhotic patients, 20 (76.9%) were anti-HBc-positive, whereas among 159 noncirrhotic ones only 31 (19.5%) were anti-HBc-positive (*P* < .0005, *x* ^2^ = 32.593).

One hundred sixty-eight patients (90.8% of the study population) tolerated well and finished the treatment schedule, while seventeen patients discontinued treatment. One hundred fifteen patients (62.1%) exhibited SVR, and 53 (28.6%) were characterized as non-SVRs. Among 115 patients with SVR, 85 (73.8%) were anti-HBc-negative, whereas among 53 non-SVRs, 32 (60.3%) were anti-HBc-negative. 

Clinical, serological, virological, and histological baseline data of the SVRs and non-SVRs groups are presented in Table 2. Overall, SVR was positively related to the presence of genotype non-1 HCV infection (*P* < .0001). In particular, compared to a genotype-1 infected patient, a patient infected with genotype 2, 3 or 4 of HCV had 4.72, 4.82 or 1.89 more chances of achieving an SVR, respectively. SVRs were younger (*P* < .0001) and had lower histological fibrosis scores (*P* < .0001), compared to non-SVRs. In the univariate analysis, we found a significant trend (30/115 versus 21/53, *P* = .057) in relationship between the anti-HBc seropositivity and the presence of non-SVR.

Using logistic regression analysis, in the multivariate model, adjusted for all baseline parameters, we found that only the advance histological stage of liver disease (*P* = .015) and the presence of genotype-1 HCV infection (*P* = .003) were independent predictors of non-SVR, whereas BMI (*P* = .451), anti-HBc seropositivity (*P* = .726) as well as patients age (*P* = .055) were not (Table 3), even taking into consideration the age subgroups (<35 years; *P* = .151, 35–55 years; *P* = .441, >55 years; *P* = .058). For patients younger than 35 years old (88.7% of whom exhibited SVR) none of the baseline parameters—neither viral genotype (*P* = .284), nor the stage of liver disease (*P* = .351)—was independent predictor of non-SVR, whereas for patients between 35–55 years old, only the presence of genotype-1 infection was independently predicted non-SVR (*P* = .008). Additionally, for patients older than 55 years only the advance histological stage of liver disease (*P* = .047), but not HCV genotype (*P* = .275), was independently predicted non-SVR (41.7% of them exhibited SVR).

The majority of patients younger than 35 years old (85.4%) were anti-HBc negative, whereas the corresponding percentages for patients 35–55 years old and for patients older than 55 years were 61.8% and 61.4%, respectively. Table 4 summarizes the clinical and histological data among anti-HBc-negative and anti-HBc-positive chronic hepatitis C patients. As we can see from this table, anti-HBc-positive patients were older and had significantly more advanced liver disease compared to anti-HBc-negative patients. Patient's age had not had an impact on the effect of the anti-HBc seropositivity in the prediction of the SVR (<35 years-*P* = .999, 35–55 years-*P* = .721, >55 years-*P* = .815), in the multivariate model that was adjusted for all the baseline parameters.

## 4. Discussion

The main finding of our study is that in treatment-naïve CHC patients with high pretreatment viral load, receiving the currently approved treatment, the advanced histological stage of liver disease and the genotype 1 infection are negatively associated with SVR achievement. Contrariwise, other baseline parameters, including the anti-HBc seropositivity, do not have impact on antiviral therapy outcome. 

Several studies suggest that the presence of detectable HBV-DNA in serum of HBsAg-negative/anti-HBc-positive patients correlates with the anti-HCV positivity [14, 18], especially in isolated anti-HBc-positive CHC patients [19] and immunocompromised individuals, such as IVDUs [15]. Moreover, it is suggested that there is an inverse correlation between the evolution of HBV-DNA and HCV-RNA levels [20]. The anti-HBc-only serology seems to represent three settings: first, false positive results with negative HBV DNA; second, recovery from a previous infection with undetectable anti-HBs titers; third, occult HBV infection. The most probable explanation for isolated anti-HBc reactivity in CHC patients with occult HBV infection is considered to be the possible interference of HCV on HBsAg synthesis [19]. In our study, we investigated the prevalence of serum HBV-DNA positivity in HBsAg-negative/anti-HBc-positive CHC patients, with or without the presence of antiHBs, and we found that only 1.97% of them exhibited detectable, low-grade, serum HBV-DNA. Our patients exhibited high pretreatment HCV-RNA (>800 IU/mL) levels, a parameter that could possibly have a significant impact in serum HBV-DNA levels. Moreover, more than 70% of patients in our cohort were prior IVDUs, a special group that has been frequently involved in occult HBV infection cases [14, 15] but only 15.6% of studied patients exhibited isolated positive anti-HBc status. The majority of them presented anti-HBc and anti-HBs seropositivity probably due to spontaneously resolved acute HBV infection in the past. This represents the common serological pattern of HBV infection among IVDUs with CHC in Greece [21]. In IVDUs with CHC, HBV-DNA is more frequent detected in liver tissue specimens (29.4%) than in patient's serum samples (1.9%), a finding that does not seem to affect their liver histopathology or the treatment outcome [15].

 Anti-HBc positivity was not related to virological-confirmed occult HBV infection in the vast majority of the CHC patients of our study. This is in accordance with the study of Haushofer et al. [22], which reported the absence of detectable serum HBV-DNA in isolated anti-HBc-positive patients with or without CHC. Moreover, anti-HBc seropositivity was not associated with the treatment outcome in the multivariate model after adjustment for the entire baseline parameters, despite a relatively significant trend that was observed in the univariate analysis. This trend was probably observed due to significant proportion of cirrhotic patients with positive anti-HBc. Previous HBV infection was linked to the advanced stage of liver disease, despite the low prevalence of occult HBV infection [23], data similar to our results.

 We studied a population relatively difficult to manage—with high pretreatment viral load and genotype-1 infection in about half of them, treated under real life conditions. Despite that, the overall SVR rates were comparable with the corresponding rates of the pivotal trials [4–6]. Several studies suggest that dose reduction and particularly premature treatment discontinuation are associated with a marked reduction of SVR rates [24, 25]. In our study, the adherence to treatment was more than 90%, and this could possibly have a substantial positive impact on the SVR rates. BMI was not an important predictor of response, as proposed [26], possibly because all patients were treated with weight-adjusted dose of pegylated-interferon a2b. Additionally, as shown in Table 1, the mean BMI of patients was 25, indicating that our cohort did not include obese subjects. Additionally, we found that the impact of the classical proposed predictors of SVR, such as viral genotype and liver histopathology, is modified according to patient's age. This finding suggests that although the patient's age is not an independent predictor of the SVR achievement and other baseline parameters also should be taken into account in our everyday clinical practice in order to predict an SVR.

Limitations of our study are the retrospective analysis of data, the lack of on-treatment or end of treatment virological data, and the absence of HBV histological data from patient's liver tissue or serum HBV-DNA data of anti-HBc-negative chronic hepatitis C patients. Also, it should be mentioned that the sensitivity of the assay, used in our study for the quantification of HBV-DNA, is 400 copies/mL. Nested PCR, which is reported to be most sensitive, is not available in our institution. Finally, up to 20% of individuals with occult hepatitis B are negative for all markers of past HBV infection aside from HBV DNA [27]. So, some cases of occult hepatitis B possibly have been missed in our trial. 

 Despite these limitations, we believe that the information from observational studies adds a pragmatic flavor of “real life data”, an aspect often missing from clinical trials. Also, it provides a basis for further research, so that preliminary observations, like that of our study, could be verified or falsified in prospective randomized control trials.

 In conclusion, anti-HBcore seropositivity does not affect the SVR achievement rate in treatment-naïve CHC patients with high pretreatment viral load, receiving a currently approved combination treatment under “real life” conditions.

## Figures and Tables

**Table 1:** Epidemiological, serological, virological, and histological baseline data of the study population. IVDU: intravenous drug users; HCV: hepatitis C virus; Anti-HBc: antibody against Hepatitis B core antigen.

Gender (male/female)	124/61
Greeks (%)	82.7
Non-Greeks (%)	17.3
IVDU (%)	70.2
HCV genotype-1 (%)	47.6
HCV genotype-2 (%)	3.6
HCV genotype-3 (%)	36.3
HCV genotype-4 (%)	12.5
Cirrhosis (%)	14.4
Anti-HBc-negative (%)	70
Anti-HBc-positive (%)	30
* *▪ Anti-HBs positive (%)	* *▪ 84.3
* *▪ HBV-DNA negative (%)	* *▪ 98.03

**Table 2:** Comparison of clinical, serological, virological, and histological baseline data among SVRs and non-SVRs. SVR: sustained virological response; BMI: body mass index; Anti-HBc: antibody against Hepatitis B core antigen (*), *P* < .05.

	SVRs	non-SVRs
	*n* = 115	*n* = 53
Age (years)	37.69 ± 11.04	50.28 ± 13.35 (*)
Gender (male/female)	73/42	34/19
BMI	24.51 ± 3.29	25.29 ± 3.17
Genotype 1 (*n*, %)	42 (36.5%)	38 (71.7%) (*)
Genotype non-1 (*n*, %)	73 (63.5%)	15 (28.3%) (*)
Grade (0–18)	5.41 ± 1.77	5.75 ± 2.02
Stage (0–6)	1.98 ± 1.12	3.33 ± 1.63 (*)
Anti-HBc (+/−)	30/85	21/32

**Table 3:** Multivariate analysis results. (*), *P* < .05; BMI: body mass index; Anti-HBc: antibody against Hepatitis B core antigen.

	Odds ratio (95% CI)	*P*-value
Gender	1.519 (0.573–4.028)	0.401
Age	0.958 (0.916–1.001)	0.055
BMI	1.060 (0.911–1.232)	0.451
Genotype (1 versus non-1)	3.550 (1.471–8.565)	0.003 (*)
Grade	1.193 (0.921–1.545)	0.181
Stage	0.625 (0.410–0.952)	0.015 (*)
Anti-HBc	1.192 (0.446–3.188)	0.726

**Table 4:** Comparison of clinical and histological baseline data among anti-HBc positive and anti-HBc negative chronic hepatitis C patients; anti-HBc: antibody against Hepatitis B core antigen.

	Anti-HBc	Anti-HBc	*P*-value
	positive	negative	
Age (years, median)	44.5	37	<.001 (*)
BMI	25.3	24.7	.873
Grade (0–18, median)	5	5	.428
Stage (0–6, median)	3	2	.005 (*)
Cirrhosis (*n*/%)	20 (76.9%)	6 (23.1%)	<.0005 (*)

(*), *P* < .05.

## References

[1] McHutchison JG, Gordon SC, Schiff ER (1998). Interferon alpha-2b alone or in combination with ribavirin as initial treatment for chronic hepatitis C. Hepatitis 
Interventional Therapy Group. *The New England Journal of Medicine*.

[2] Lai MY, Kao JH, Yang PM (1996). Long-term efficacy of ribavirin plus interferon alpha in the treatment of chronic hepatitis C. *Gastroenterology*.

[3] National Institutes of Health (2002). NIH consensus statement on management of hepatitis C: 2002. *NIH Consensus and State-of-the-Science Statements*.

[4] Hadziyannis SJ, Sette H, Morgan TR (2004). Peginterferon-*α*2a and ribavirin combination 
therapy in chronic hepatitis C: a randomized study of treatment duration and ribavirin dose. *Annals of Internal Medicine*.

[5] Manns MP, McHutchison JG, Gordon SC (2001). Peginterferon alpha-2b plus ribavirin compared with interferon alpha-2b plus ribavirin for initial treatment of chronic hepatitis C: a randomised trial. *The Lancet*.

[6] Fried MW, Shiffman ML, Reddy KR (2002). Peginterferon alpha-2a plus ribavirin for chronic hepatitis C virus infection. *The New England Journal of Medicine*.

[7] Heathcote EJ, Shiffman ML, Cooksley WGE (2000). Peginterferon alpha-2a in patients with chronic hepatitis C and cirrhosis. *The New England Journal of Medicine*.

[8] Ganem D, Prince AM (2004). Hepatitis B virus infection—natural history and clinical consequences. *The New England Journal of Medicine*.

[9] Chemin I, Trépo C (2005). Clinical impact of occult HBV infections. *Journal of Clinical Virology*.

[10] Hui C-K, Lau E, Wu H (2006). Fibrosis progression in chronic hepatitis C patients with occult hepatitis B co-infection. *Journal of Clinical Virology*.

[11] Fukuda R, Ishimura N, Niigaki M (1999). Serologically silent hepatitis B virus co-infection in patients with hepatitis C virus-associated 
chronic liver disease: clinical and virological significance. *Journal of Medical Virology*.

[12] Vitale F, Tramuto F, Orlando A (2008). Can the serological status of “anti-HBc alone” be considered a sentinel marker for 
detection of “occult” HBV infection?. *Journal of Medical Virology*.

[13] Sagnelli E, Coppola N, Scolastico C, Mogavero AR, Filippini P, Piccinino F (2001). HCV genotype and “silent” HBV co-infection: two main risk factors for a more severe liver disease. *Journal of Medical Virology*.

[14] Cacciola I, Pollicino T, Squadrito G, Cerenzia G, Orlando ME, Raimondo G (1999). Occult hepatitis B virus infection in patients with chronic hepatitis C liver disease. *The New England Journal of Medicine*.

[15] Fabris P, Brown D, Tositti G (2004). Occult hepatitis B virus infection does not affect liver histology or response to therapy with interferon alpha and ribavirin in intravenous drug users with chronic hepatitis C. *Journal of Clinical Virology*.

[16] Carreño V, Bartolomé J, Castillo I, Quiroga JA (2008). Occult hepatitis B virus and hepatitis C virus infections. *Reviews in Medical Virology*.

[17] Ishak K, Baptista A, Bianchi L (1995). Histological grading and staging of chronic hepatitis. *Journal of Hepatology*.

[18] Drosten C, Nippraschk T, Manegold C (2004). Prevalence of hepatitis B virus DNA in anti-HBc-positive/HBsAg-negative sera correlates with HCV 
but not HIV serostatus. *Journal of Clinical Virology*.

[19] Weber B, Melchior W, Gehrke R, Doerr HW, Berger A, Rabenau H (2001). Hepatitis B virus markers in anti-HBc only positive individuals. *Journal of Medical Virology*.

[20] Khattab E, Chemin I, Vuillermoz I (2005). Analysis of HCV co-infection with occult hepatitis B virus in patients undergoing IFN therapy. *Journal of Clinical Virology*.

[21] Elefsiniotis IS, Pantazis KD, Ketikoglou ID, Koutsounas SI, Tsianos EV (2006). Changing serological status and low vaccination-induced protection rates against 
hepatitis B characterize chronic hepatitis C virus-infected injecting drug users in Greece: need for immunization policy. *European Journal of Gastroenterology & Hepatology*.

[22] Haushofer AC, Hauer R, Brunner H (2004). No evidence of hepatitis B virus activity in patients with anti-HBc antibody positivity with or without anti-hepatitis C virus antibody positivity. *Journal of Clinical Virology*.

[23] Giannini E, Ceppa P, Botta F (2003). Previous hepatitis B virus infection is associated with worse disease stage and occult hepatitis B virus infection has low prevalence and pathogenicity in hepatitis C virus-positive patients. *Liver International*.

[24] McHutchison JG, Manns M, Patel K (2002). Adherence to combination therapy enhances sustained response in genotype-1-infected patients with 
chronic hepatitis C. *Gastroenterology*.

[25] Manns MP (2004). Adherence to combination therapy: influence on sustained virologic response and economic impact. *Gastroenterology Clinics of North America*.

[26] Tarantino G, Conca P, Sorrentino P, Ariello M (2006). Metabolic factors involved in the therapeutic response of patients with hepatitis C virus-related 
chronic hepatitis. *Journal of Gastroenterology and Hepatology*.

[27] Torbenson M, Thomas DL (2002). Occult hepatitis B. *Lancet Infectious Diseases*.

